# Efficacy of AI Models in Detecting Heart Failure Using ECG Data: A Systematic Review and Meta-Analysis

**DOI:** 10.7759/cureus.78683

**Published:** 2025-02-07

**Authors:** Salman Khan, Komal Qayyum, Abdul Qadeer, Maria Khalid, Somaan Anthony, Wafa khan, Moula Ghulam, Zainab Jamil, Nouman Anthony

**Affiliations:** 1 Cardiology, Rehman Medical Institute, Peshawar, PAK; 2 Cardiology, Northwest General Hospital and Research Centre, Peshawar, PAK; 3 Cardiology, Khyber Medical University Institute of Medical Sciences (KMU-IMS), Peshawar, PAK; 4 Medicine, Lady Reading Hospital Peshawar, Peshawar, PAK; 5 Medicine, Rehman Medical Institute, Peshawar, PAK; 6 General Medicine, Rehman Medical Institute, Peshawar, PAK

**Keywords:** ai and cardiovascular disease, ai and ecg, ai and heart failure, ai and robotics in healthcare, efficacy of ai in cardiac medicine

## Abstract

Heart failure (HF) is the most common cause of death worldwide, characterized by low ejection fraction, substantial mortality, morbidity, and poor quality of life. Recent advancements in artificial intelligence (AI) present a promising avenue for enhancing diagnostic precision, particularly in the analysis of electrocardiogram (ECG) data. This systematic review and meta-analysis aim to synthesize current evidence on the diagnostic performance of AI models in detecting HF using ECG data. PubMed and Google Scholar databases were systematically searched from inception up to July 1, 2023, to identify original articles assessing the predictive ability of AI in HF diagnosis. A total of 218,202 participants were included, with individual studies ranging from 59 to 110,000 participants. The pooled sensitivity, specificity, and diagnostic odds ratio (DOR) for the 13 included studies, with a 97.5% confidence interval (CI), were 0.93 (CI: 0.81-0.98), 0.95 (CI: 0.89-0.97), and 303.65 (CI: 53.12-1734), respectively. The sensitivity and specificity ranged from 0.12 to 1.00 and 0.66 to 1.00, respectively, indicating substantial variability in AI model performance, which may impact their generalizability and clinical reliability. AI-based algorithms utilizing ECG data are a reliable, accurate, and promising tool for the screening, detection, and monitoring of HF. However, further prospective studies are needed, particularly randomized controlled trials and large-scale longitudinal studies across diverse populations, to evaluate the long-term clinical impact, generalizability, and real-world applicability of these AI-driven diagnostic tools.

## Introduction and background

Cardiovascular diseases remain a leading cause of morbidity and mortality worldwide, with heart failure (HF) emerging as a significant public health concern. Globally, HF affects an estimated 64 million individuals [1]. Timely and accurate diagnosis is paramount for effective management and improved patient outcomes. HF is a complex clinical syndrome marked by symptoms and signs resulting from structural or functional impairment of ventricular filling or ejection of blood. In congestive heart failure (CHF), patients commonly present with fatigue, dyspnea, reduced exercise tolerance, and systemic or pulmonary congestion [2]. Left ventricular ejection fraction (LVEF) plays a crucial role in classifying HF, with HF with reduced ejection fraction (HFrEF) typically defined as LVEF ≤35% or ≤40% [3]. Diagnosis relies on a combination of clinical symptoms, physical examination, and diagnostic tests, including electrocardiogram (ECG), echocardiography, and biomarkers [3]. Among these, electrocardiography remains a cornerstone for assessing cardiac function, capturing the heart’s electrical activity, and offering valuable diagnostic insights.

As artificial intelligence (AI) continues to be integrated into medical practice, there is a growing body of literature investigating its potential to detect HF through ECG data. AI techniques such as deep learning, convolutional neural networks (CNNs), and support vector machines (SVMs) have demonstrated promising capabilities in identifying subtle patterns and anomalies within ECG recordings, potentially improving diagnostic accuracy [4]. Several studies have evaluated AI-driven ECG analysis for HF detection [4-6]. However, existing literature presents several challenges and inconsistencies, including variability in data sources (public datasets vs. patient-level data), lack of external validation, and limited generalizability across different populations. Many AI models are trained and tested in high-resource clinical settings, yet their performance in low-resource settings, ethnically diverse populations, and primary care environments remains underexplored. Moreover, inconsistencies in the reported diagnostic performance of AI models raise concerns about their real-world applicability and clinical decision-making impact [7].

This systematic review meticulously examines a comprehensive array of studies that have delved into the intersection of AI and ECG data for HF detection. By synthesizing the available evidence through a rigorous meta-analysis, we aim to provide a quantitative overview of the diagnostic accuracy of ECG-based AI models for the diagnosis of HF. This research's significance lies in its potential to inform clinical practice and contribute to the ongoing discourse surrounding the integration of AI into mainstream healthcare. Understanding the strengths and limitations of AI models in HF detection is essential for clinicians, researchers, and policymakers as they navigate the evolving landscape of cardiovascular diagnostics. Through this systematic review and meta-analysis, we endeavor to distill the current state of knowledge, identify gaps in understanding, and provide a foundation for future research endeavors to optimize AI's role in cardiovascular medicine.

## Review

Materials and methods

Study Selection

The study selection process was guided by the Participants, Index test(s), Reference standard, and Diagnostic outcomes (PIRD) criteria, ensuring a systematic and transparent approach to identifying eligible studies. Participants included individuals diagnosed with CHF and reduced LVEF of <40%, irrespective of age, gender, or comorbid conditions. Studies exclusively focused on pediatric populations, animal models, or non-HF cardiac conditions were excluded. AI algorithms using ECG data served as the index test for screening and diagnosing HF. Various AI models, including machine learning and deep learning approaches, were assessed. Studies without a clear description of the AI model or those lacking a comparison with standard diagnostic methods were excluded. The reference standard included transthoracic echocardiogram (TTE), biochemical markers (e.g., BNP/NT-proBNP), and symptom-based clinical diagnosis. Only studies that used at least one established reference standard to confirm HF diagnosis were included, ensuring diagnostic accuracy. The primary diagnostic outcomes extracted from studies were sensitivity, specificity, and diagnostic odds ratio (DOR). Data were collected based on a confusion matrix, including true positives (TPs), false positives (FPs), true negatives (TNs), and false negatives (FNs) [8].

Studies with partial data or missing key diagnostic accuracy metrics were carefully evaluated. If relevant performance metrics could be extracted through available data or supplementary materials, the study was retained. However, if data essential for constructing a 2 × 2 confusion matrix (i.e., TP, FP, TN, FN) were incomplete or could not be inferred, the study was excluded from the meta-analysis. Discrepancies in study selection were resolved by independent reviewers, ensuring consistency and methodological rigor.

Search Strategy

A comprehensive literature search for original articles on the ability of AI to predict HF based on ECG signals was conducted using PubMed (including Medline) and Google Scholar. The initial search was performed on May 13, 2020, and the search was systematically updated until July 1, 2023, to ensure the inclusion of recent studies. The update was conducted to capture newly published literature, reflecting advancements in AI-based diagnostic models. The search strategy utilized the following Boolean query: ((heart failure) OR (left ventricle dysfunction)) AND ((electrocardiogram) OR (ECG)) AND ((Machine Learning) OR (Artificial Intelligence) OR (Neural Networks) OR (Support Vector Machine) OR (Naive Bayes)), with searches restricted to titles and abstracts. To maximize inclusivity, we tested additional synonyms and alternative terms such as “cardiac dysfunction,” “deep learning,” and “random forest,” but their inclusion did not significantly alter search results or yield relevant new studies.

To supplement the database search, a bibliographic review was conducted using citation tracking and reference screening. Specifically, we examined the reference lists of all included studies to identify additional relevant articles that met our eligibility criteria. Forward citation tracking was also performed using Google Scholar to identify subsequent studies that cited key included articles. Studies identified through these methods underwent the same eligibility screening process. The detailed Medical Subject Headings (MeSH) approach for PubMed and Google Scholar is presented in Table 1.

**Table 1:** The search strategy, search engines used, and the number of results displayed

Database	Search strategy	N
PubMed	((heart failure) OR (left ventricle dysfunction))	357639
	((heart failure) OR (left ventricle dysfunction)) AND ((ECG) OR (electrocardiogram))	32311
	((heart failure) OR (left ventricle dysfunction)) AND ((ECG) OR (electrocardiogram)) AND ((Machine Learning) OR (Artificial Intelligence) OR (Neural Networks) OR (Support Vector Machine) OR (Naive Bayes))	272
Google Scholar	((heart failure) OR (left ventricle dysfunction)) AND ((ECG) OR (electrocardiogram)) AND ((Machine Learning) OR (Artificial Intelligence) OR (Neural Networks) OR (Support Vector Machine) OR (Naive Bayes))	2620

**Figure 1:**
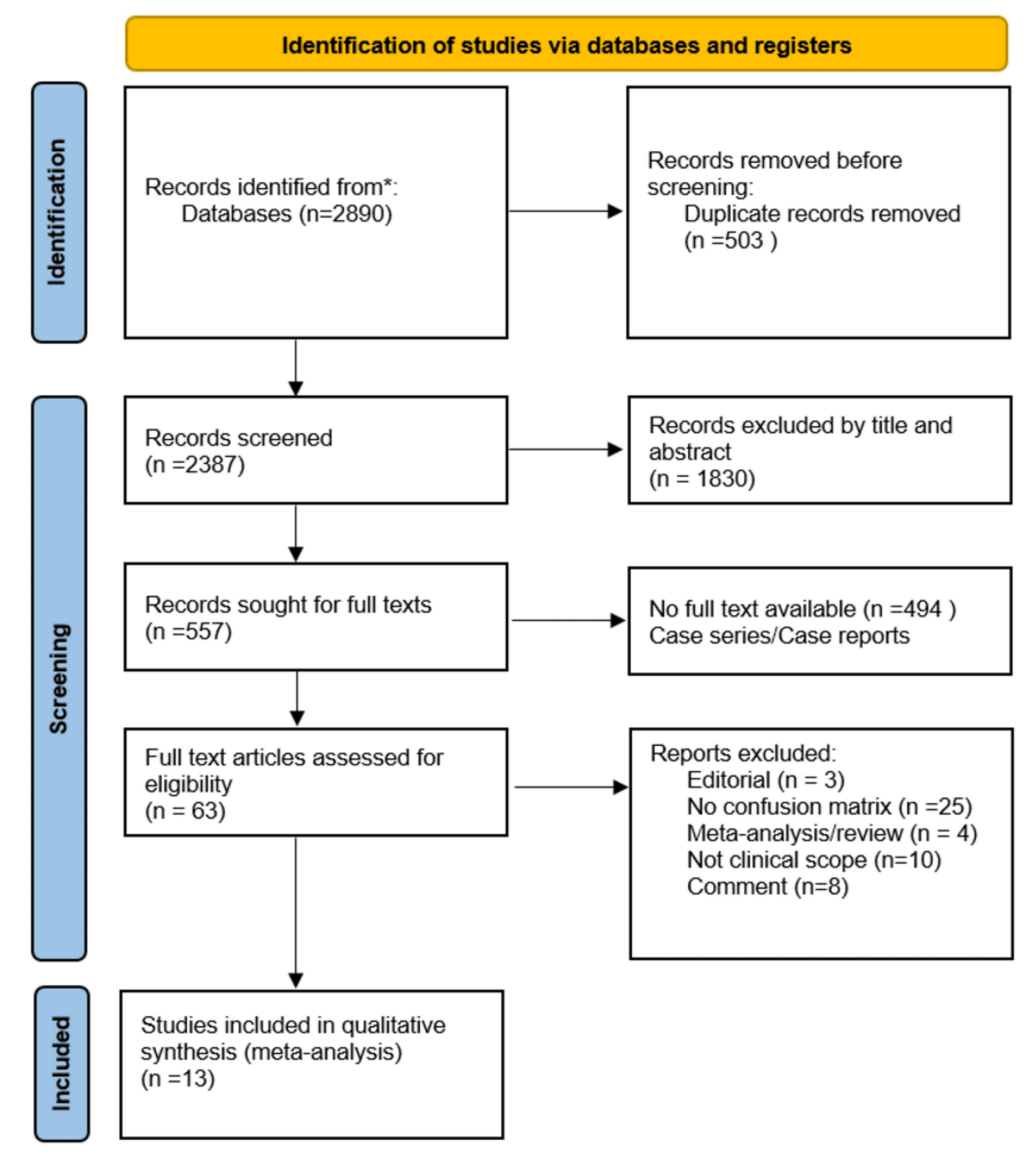
The flowchart summarizing the literature screening and study selection process *: PubMed and Google Scholar

The authors independently reviewed and performed the literature search and title/abstract screening. They excluded articles not associated with the research topic. The remaining articles were screened for eligibility by analyzing the full text. An independent reviewer resolved disagreements and recorded the reasons for exclusion. The literature search results are shown in the Preferred Reporting Items for Systematic Reviews and Meta-Analyses (PRISMA) flowchart (Figure 1).

Eligibility Criteria

The inclusion criteria for this systematic review were defined as studies involving people with CHF and reduced LVEF of <40%. The index test used in the studies had to be an AI algorithm analyzing ECG data. Studies were included if they reported sufficient data for a planned meta-analysis, specifically the diagnostic accuracy performance metrics such as TPs, FPs, TNs, and FNs, or if these metrics could be extracted from the study. Studies that covered all genders were eligible, and only free-access papers were considered for inclusion.

The exclusion criteria involved animal studies and methodological studies that solely focused on explaining programming details without any diagnostic application. Additionally, letters, editorials, conference abstracts, systematic reviews, meta-analyses, consensus statements, and guidelines were excluded. Studies that did not provide sufficient data to construct a 2 × 2 confusion matrix (i.e., TP, FP, TN, and FN) were also excluded. Articles that required purchase or paid access were not considered. Lastly, papers focusing on cardiac pathologies other than HF as the outcome measure were excluded from the analysis.

Data Extraction and Study Quality Assessment

The extracted data included study characteristics such as author names, year of publication, sample sizes, ECG data types (e.g., heart rate variability, raw ECG signals), machine learning (ML) models used, dataset types (public or patient level), definitions of HF, and country of origin. Studies with sample sizes smaller than 1000 were excluded from sensitivity analysis. Hierarchical summary receiver operating characteristic (HSROC) curves were plotted to visualize disease prevalence, sensitivity, and specificity with 95% confidence intervals (CIs).

To assess study quality and risk of bias, we applied the Quality Assessment of Diagnostic Accuracy Studies-2 (QUADAS-2) tool, as it remains the most widely used framework for evaluating diagnostic accuracy studies. Although originally designed for traditional diagnostic tests, QUADAS-2 was adapted for AI-based studies by assessing key domains: patient selection, index test (AI algorithm), reference standard (e.g., echocardiography), and flow/timing. Given the limitations of QUADAS-2 for AI research, alternative tools such as the Prediction Model Risk of Bias Assessment Tool (PROBAST) and Methodological Index for Nonrandomized Studies (MINORS) could be considered for future evaluations of AI-based predictive models. Clinical applicability was assessed based on whether (1) study populations reflected real-world HF cases, (2) AI models used clinically relevant ECG data, and (3) validated reference standards were employed, with concerns rated as low, high, or unclear.

Two independent reviewers performed data extraction and risk assessments, with discrepancies resolved through discussion or arbitration by a third reviewer. To measure inter-reviewer agreement, Cohen’s kappa statistic was calculated, with a kappa value of ≥0.80 indicating excellent agreement. By employing QUADAS-2 while acknowledging its limitations, we aimed to provide a structured assessment of study quality, highlighting the need for AI-specific risk assessment frameworks in future research.

Statistical Analysis

We performed a diagnostic test meta-analysis using a random-effects model to calculate pooled sensitivity, specificity, and DOR, all with 95% confidence intervals (CIs). Forest plots for sensitivity and specificity were generated using the inverse variance method, with weights assigned based on study precision and sample size. The metaDTA tool in R was used for statistical computations. To assess heterogeneity, we calculated I², which quantifies the percentage of variability due to heterogeneity rather than chance, and τ², which estimates between-study variance in diagnostic accuracy.

A hierarchical summary HSROC model was used to generate the SROC curve, which provides a comprehensive visualization of the diagnostic performance by plotting sensitivity against 1-specificity. Each study was represented as a circle, with size proportional to its weight, while the summary estimate was shown as a rectangle, surrounded by its 95% confidence region. The HSROC curve is particularly useful in diagnostic meta-analyses as it accounts for variations in test thresholds across studies and helps in assessing the overall discriminative ability of AI-based ECG models for detecting HF.

Sensitivity analysis was performed by excluding studies with sample sizes <1000 and by stratifying results based on the type of ML model used. This allowed us to evaluate the robustness of our pooled estimates and the potential impact of study size on diagnostic accuracy. By incorporating these methods, we aimed to provide a rigorous and transparent assessment of the diagnostic utility of AI-based ECG models in HF detection.

Results

The search strategy yielded 2890 studies. After removing duplicates, 2387 articles were eligible for full-text review. We excluded 50 studies due to insufficient data to create a confusion matrix for our meta-analysis, leading to 13 articles in the final analysis. Reasons for exclusion were recorded. The PRISMA chart for our review is shown in Figure 1.

Study Characteristics

A total of 218,202 participants were included in this meta-analysis, with individual study sample sizes ranging from 59 to 110,000 participants. Five studies were conducted using public ECG datasets, while eight used patient-level datasets. The distinction between these datasets is important, as public datasets may introduce selection bias due to preprocessed data, whereas patient-level datasets reflect real-world clinical variability and may enhance generalizability. Ten out of the 13 included studies were conducted in American centers [9-19], which raises concerns about the generalizability of findings to non-Western populations. This geographical concentration may limit the applicability of AI models to diverse ethnic and demographic groups, necessitating future studies in underrepresented regions.

Different AI models were employed across studies, including support vector machines (SVM), K-nearest neighbor (KNN), and convolutional neural networks (CNN). The CNN-based AI ECG model, originally developed by the Mayo Clinic, was the most frequently used algorithm in five studies [14,15,18-20]. CNNs are particularly advantageous in ECG analysis due to their ability to automatically extract and learn hierarchical features from raw ECG waveforms, reducing the need for manual feature engineering compared to classical ML models such as SVM or KNN. However, the reuse of similar CNN models and overlapping datasets in some studies could introduce potential bias, affecting the independence of included studies. To mitigate this, we ensured that our meta-analysis included only studies with distinct patient populations or explicitly reported independent datasets.

The included studies analyzed various ECG data formats, including raw ECG, ECG images, ECG signals, heart rate variability (HRV), and ECG-enabled stethoscope signals. Eight studies had sample sizes smaller than 1000 [9,10,13,16,18,20], while the remaining had larger cohorts, which may influence statistical power and robustness. The extracted confusion matrix data is presented in Table 2, while detailed study characteristics are summarized in Table 3.

**Table 2:** Summary of studies included in the meta-analysis CML: classical machine learning; CHF: congestive heart failure; LVSD: left ventricular systolic dysfunction; EF: ejection fraction

Author	Year	Study design	Type of AI model	ECG data	Broad AI model classification	Dataset	Definition of heart failure	Validation data set	Country of Origin
Pecchia et al., 2011 [9]	2011	Retrospective cohort	Classification and regression tree (CART) method	HRV	cML	Public	CHF	No	America
Chen et al., 2016 [10]	2016	Retrospective cohort	Decision tree-support vector machine (SVM)	HRV	cML	Public	CHF	No	America
Acharya et al., 2016 [11]	2016	Retrospective cohort	Support vector machine (SVM)	HRV	cML	Public	CHF	No	America
Acharya et al., 2018 [12]	2018	Retrospective cohort	Convolutional neural network (CNN)	ECG signals	DL	Public	CHF	No	America
Attia et al., 2019 [13]	2019	Retrospective cohort	Convolutional neural network (CNN)	Raw ECG	DL	Patient-level	LVSD, EF < 35%	Yes	America
Attia et al., 2019 [14]	2019	Prospective cohort	Convolutional neural network (CNN)	ECG signals	DL	Patient-level	LVSD, EF < 35%	Yes	America
Hussain et al., 2020 [16]	2020	Retrospective cohort	Support vector machine (SVM), decision tree (DT), k-nearest neighbor (KNN)	HRV	cML	Public	CHF	No	America
Adedinsewo et al., 2020 [15]	2020	Retrospective cohort	AI-ECG algorithm (CNN)	Raw ECG	DL	Patient-level	LVSD	Yes	America
Attia et al., 2021 [17]	2021	Retrospective cohort	AI-ECG (CNN)	Raw ECG	DL	Patient-level	LVSD	yes	America
Harmon et al., 2022 [19]	2022	Retrospective cohort	AI-ECG (CNN)	ECG signals	DL	Patient level	LVSD, EF<40%	Yes	America
Bachtiger et al., 2022 [20]	2022	Prospective cohort	AI-ECG (CNN)	ECG-enabled stethoscope signals	DL	Patient level	LVSD < 40% EF	Yes	UK
Attia et al., 2022 [18]	2022	Prospective cohort	AI-ECG (CNN)	ECG-enabled stethoscope signals	DL	Patient level	LVSD, EF<35%,40% or <50%	No	America
Surendra et al., 2023 [21]	2023	Prospective cohort	Convolutional neural network (CNN)	ECG signals	DL	Patient level	CHF	yes	Germany

**Table 3:** Study-level data of confusion matrices TP: true positive; FN: false negative; FP: false positive; TN: true negative

Author	Year	TP	FN	FP	TN	Total subjects(N)	Sensitivity	Specificity
Pecchia et al., 2011 [9]	2011	26	3	0	54	83	89.70%	100.00%
Chen et al., 2016 [10]	2016	36	1	0	22	59	97.30%	100.00%
Acharya et al., 2016 [11]	2016	779	24	15	840	1658	97.00%	98.20%
Acharya et al., 2018 [12]	2018	29660	340	794	79206	110000	98.90%	99.00%
Attia et al., 2019 [13]	2019	3567	564	6977	41762	52870	86.30%	85.70%
Attia et al., 2019 [14]	2019	73	13	96	340	522	84.90%	78.00%
Hussain et al., 2020 [16]	2020	39	3	5	69	116	92.90%	93.20%
Adedinsewo et al., 2020 [15]	2020	121	43	183	1259	1606	73.80%	87.30%
Attia et al., 2021 [17]	2021	7	19	111	4140	4277	26.90%	97.40%
Harmon et al., 2022 [19]	2022	1848	0	2080	41058	44986	100.00%	95.20%
Bachtiger et al., 2022 [20]	2022	67	14	157	626	864	82.70%	79.90%
Attia et al., 2022 [18]	2022	7	0	0	93	100	100.00%	100.00%
Surendra et al., 2023 [21]	2023	43	21	307	690	1061	67.20%	69.20%

Diagnostic Performance of HF

**Figure 2:**
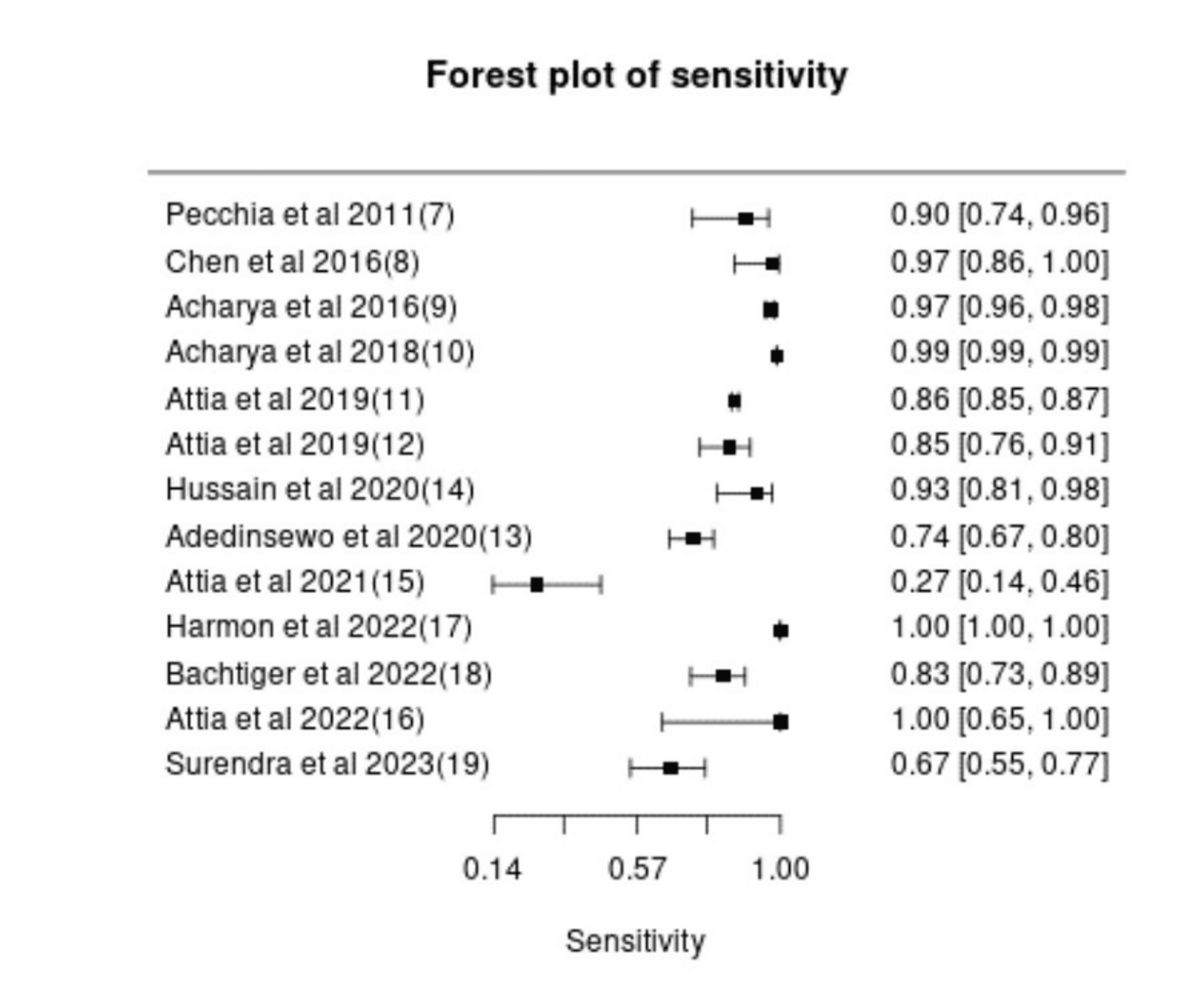
Forest plot of pooled sensitivity

**Figure 3:**
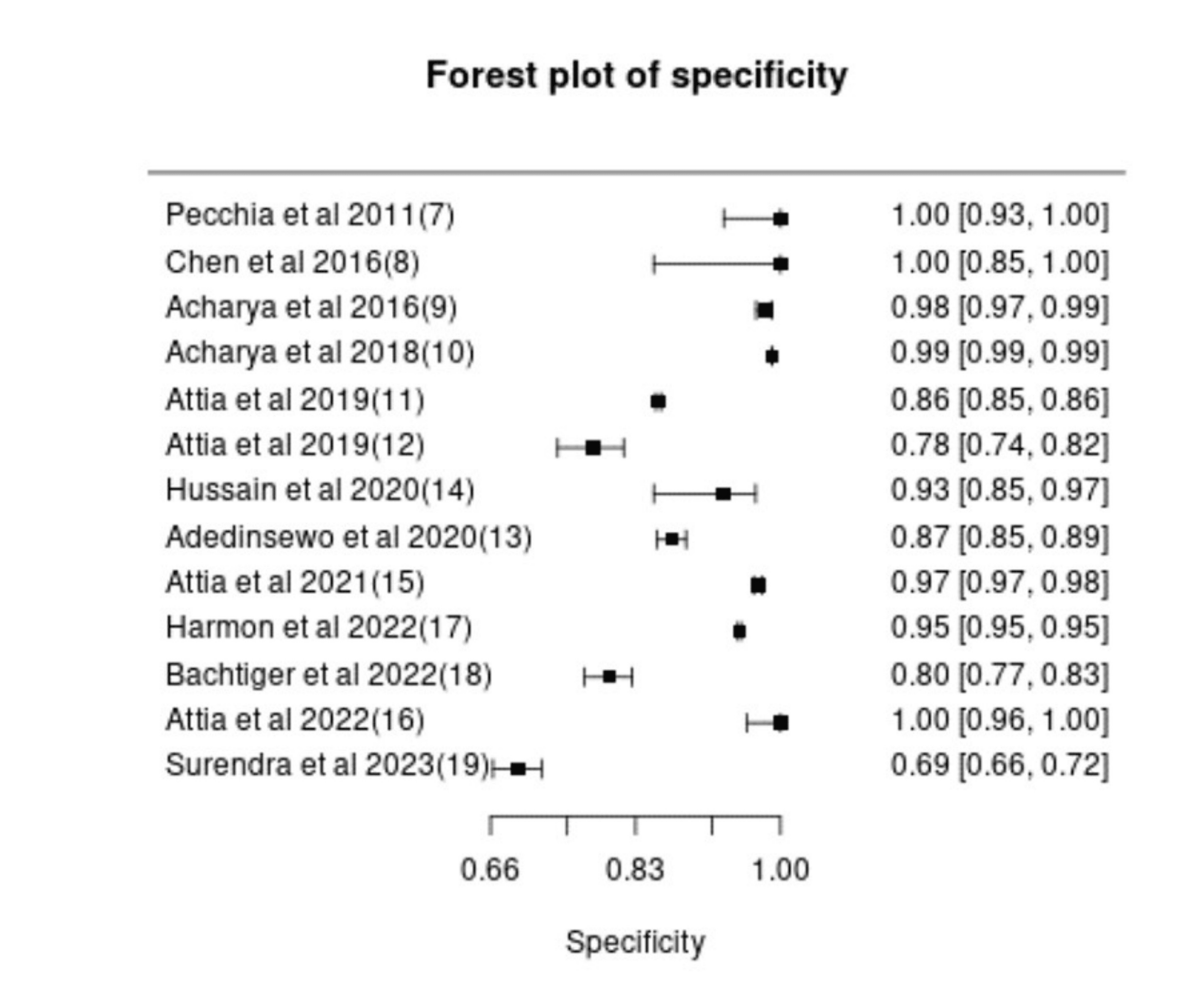
Forest plot of specificity

The pooled sensitivity, specificity, and DOR for all 13 included studies with 97.5% CI were 0.93 (CI: 0.81-0.98), 0.95 (CI: 0.89-0.97), and 303.6 (CI: 53.1-1734), respectively. These results suggest that AI models exhibit strong diagnostic performance for HF detection using ECG data, with high sensitivity ensuring minimal missed cases and high specificity reducing FP. However, the sensitivity and specificity varied widely across studies (0.12-1.00 and 0.66-1.00, respectively), likely due to differences in dataset sizes, population characteristics, and AI model types. The forest plots for pooled sensitivity and specificity are shown in Figures 2, 3. Studies using larger datasets and convolutional neural networks (CNNs) generally demonstrated higher accuracy, whereas smaller studies or those using classical ML models (e.g., SVM, KNN) exhibited greater variability in diagnostic performance.

**Figure 4:**
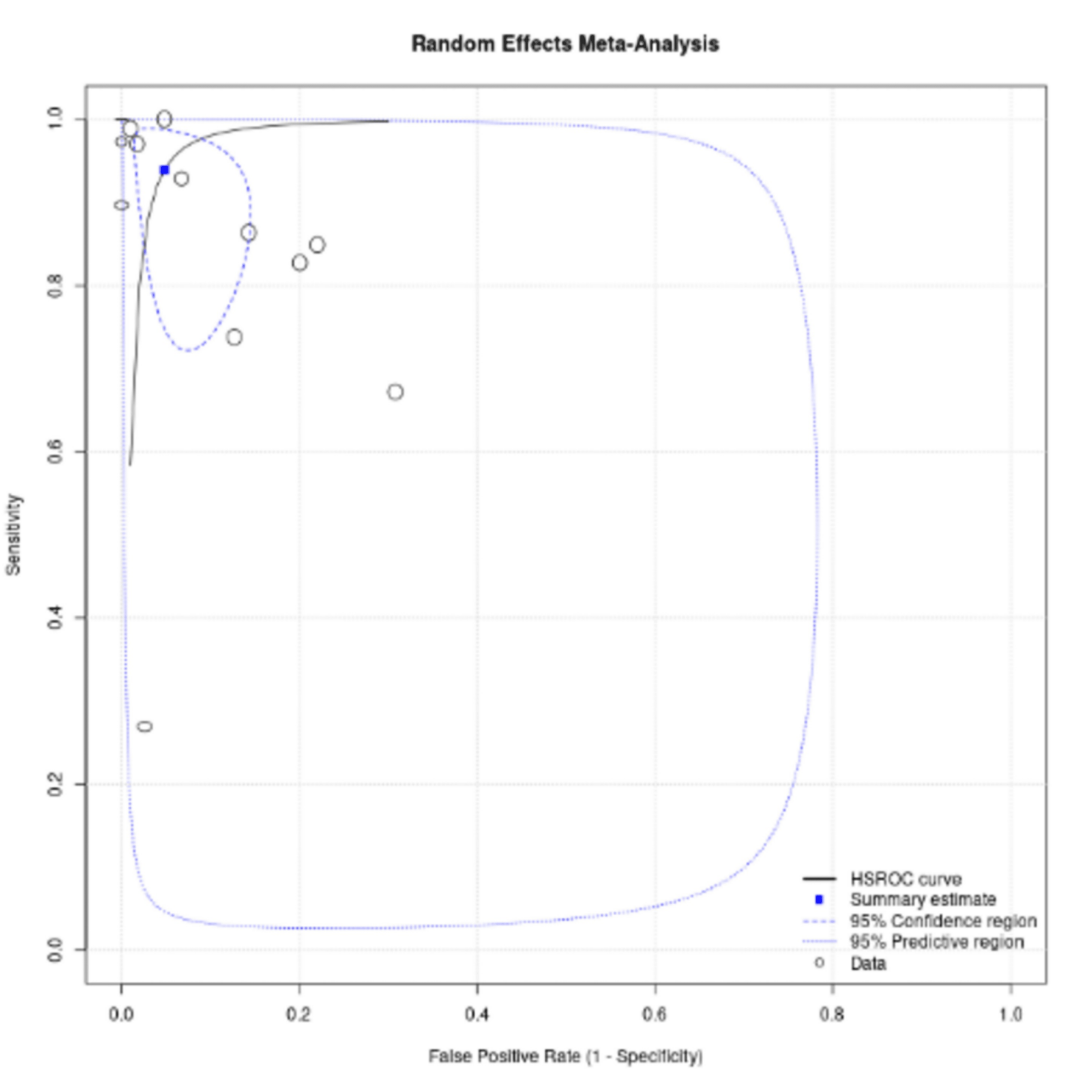
Sensitivity analysis with exclusion of studies with a sample size of <1000

Figure 4 shows the HSROC curve of AI based on an ECG for HF diagnosis. Each circle represents an individual study, with the sizes of the circle proportional to the study size. The rectangle represents summary sensitivity and specificity, and the curve represents the 95% confidence intervals.

**Figure 5:**
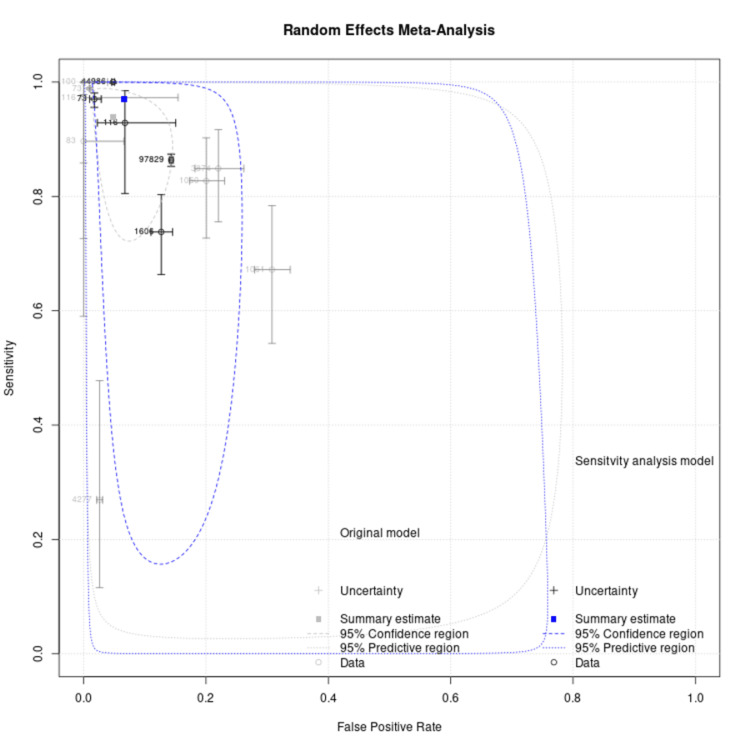
The HSROC curve with sensitivity analysis HSROC: the hierarchical summary receiver operating characteristic

**Figure 6:**
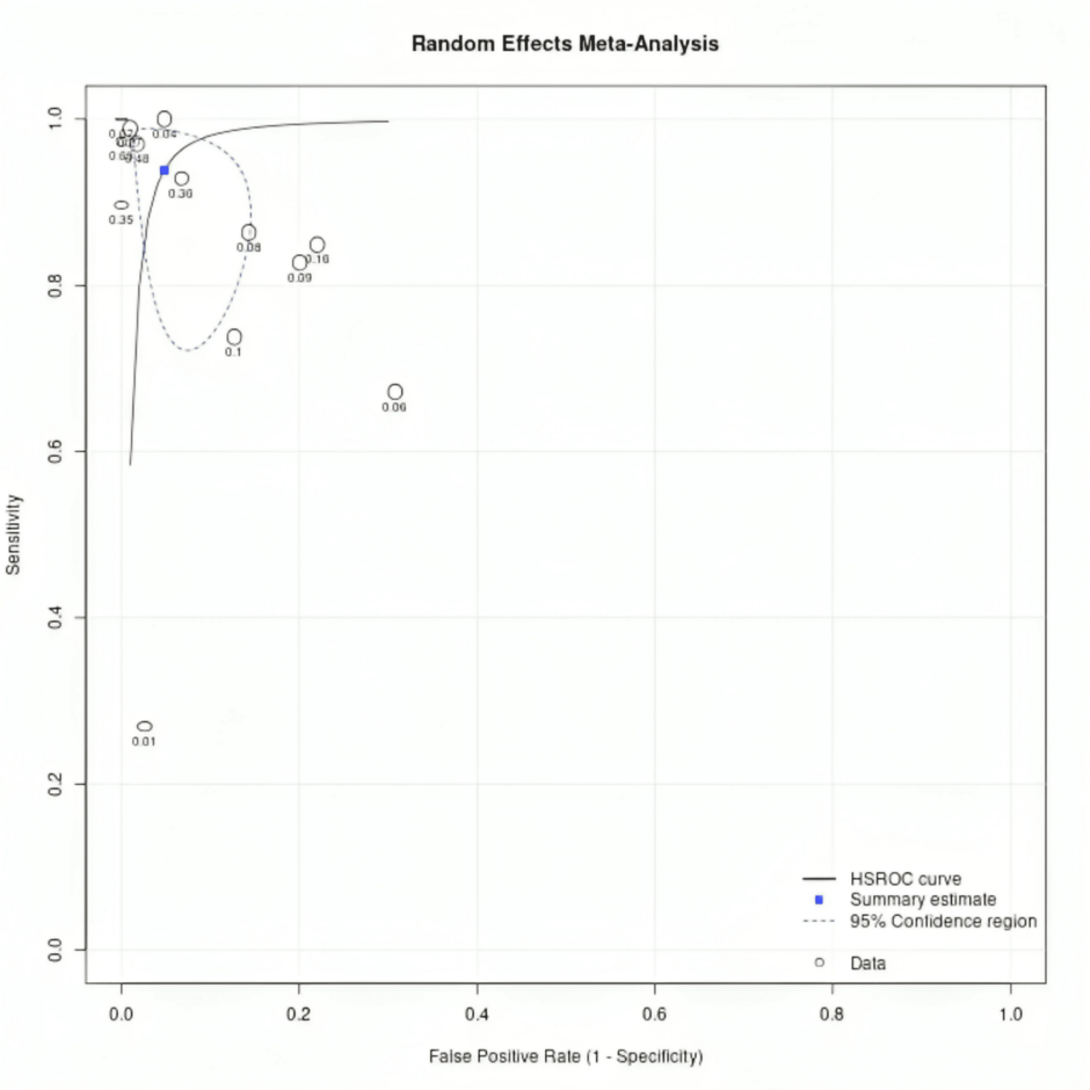
HSROC curve representing disease prevalence

The HSROC curve (Figure 5) illustrates the trade-off between sensitivity and specificity across studies while accounting for variability in diagnostic thresholds. Larger studies contributed more weight to the pooled estimate, as shown by the size of the circles, while Figure 6 highlights disease prevalence across included studies, which can influence model performance. The confusion matrix data provide deeper insight into practical diagnostic accuracy, with higher true positive rates in CNN-based models compared to classical ML approaches. These findings suggest that AI-driven ECG analysis could serve as a reliable noninvasive screening tool, but its implementation in clinical practice would benefit from further external validation in diverse populations.

Sensitivity analysis, performed by excluding studies with sample sizes of <1000, resulted in a more stable HSROC curve (Figure 5) and slightly improved pooled specificity (0.98 vs. 0.95) and DOR (534.2 vs. 303.6). This indicates that smaller studies may have introduced variability in diagnostic accuracy, potentially due to overfitting or limited generalizability. However, the overall conclusions remain consistent, reinforcing the high diagnostic potential of AI-based ECG models for HF detection.

Sensitivity analysis was performed by excluding the studies with sample sizes less than 1000, using sample size as a covariate with the resulting HSROC curve, as shown in Figure 5. After sensitivity analysis and 97.5 % CI, the specificity and DOR were 0.98 (0.93-0.95) and 534.2 (76.4-3731.3), respectively. Figure 5 shows the HSROC curve with sensitivity analysis. ROC curve showing summary data points, summary estimate, 95% confidence region, 95% predictive region, and HSROC curve for all studies, excluding studies with a sample size of <1000. The sample size of each study is mentioned with the circle, and the sensitivity and specificity of each study are represented by two lines passing through each circle. Estimates from studies in sensitivity analysis are in black, and the overall pooled estimate is in blue. Figure 5 shows the HSROC curve with sensitivity analysis.

Risk of Bias

Overall, the studies were relatively well-reported, but several biases were identified that could impact study outcomes and generalizability. As shown in Figure 6, concerns about patient selection bias were frequently marked as "unclear." This was primarily due to insufficient details on inclusion criteria, the potential selection of high-risk patients, and the exclusion of asymptomatic or undiagnosed cases, which could limit the applicability of AI models to broader populations. For example, studies that included only patients undergoing echocardiography might have introduced spectrum bias, leading to an overestimation of AI performance in real-world screening. Future research should ensure clear documentation of inclusion/exclusion criteria, include diverse patient populations, and assess AI models in both symptomatic and asymptomatic cohorts to improve generalizability.

A high risk of bias was noted for the index test and reference standard in several studies, largely due to unclear AI model thresholds, lack of external validation, and inconsistent reference standards. Many studies relied on echocardiography (EF <40%) as a reference standard, but variations in echocardiographic techniques and operator-dependent factors may have affected consistency. Additionally, some AI models were trained and validated on the same dataset without proper external validation, increasing the risk of overfitting. The lack of standardization in ML model reporting, particularly regarding threshold optimization and preprocessing methods, remains a common issue in AI research. Future studies should clearly define diagnostic thresholds, perform independent external validation, and use standardized ML reporting guidelines (e.g., TRIPOD-AI) to improve reproducibility and reduce bias.

The flow and timing domain was marked as low risk in most studies, indicating that the diagnostic process and outcome assessments were performed in a logical and clinically relevant manner. This strengthened the findings, as it ensured that outcome assessments were not influenced by retrospective data manipulation. However, this uniformity may have masked other biases, such as dataset drift (AI models trained on historical ECG data may perform differently on newer populations) or limited follow-up periods affecting long-term diagnostic accuracy. The pooled sensitivity and specificity estimates may have been influenced by selection bias and variability in AI model thresholds, underscoring the need for prospective validation studies with real-time AI implementation in diverse clinical settings. Addressing these biases in future research will require clearer patient selection criteria, better reporting of ML model thresholds, and comprehensive external validation to ensure AI models translate effectively to real-world practice. Figure 6 shows the HSROC curve representing disease prevalence.

**Figure 7:**
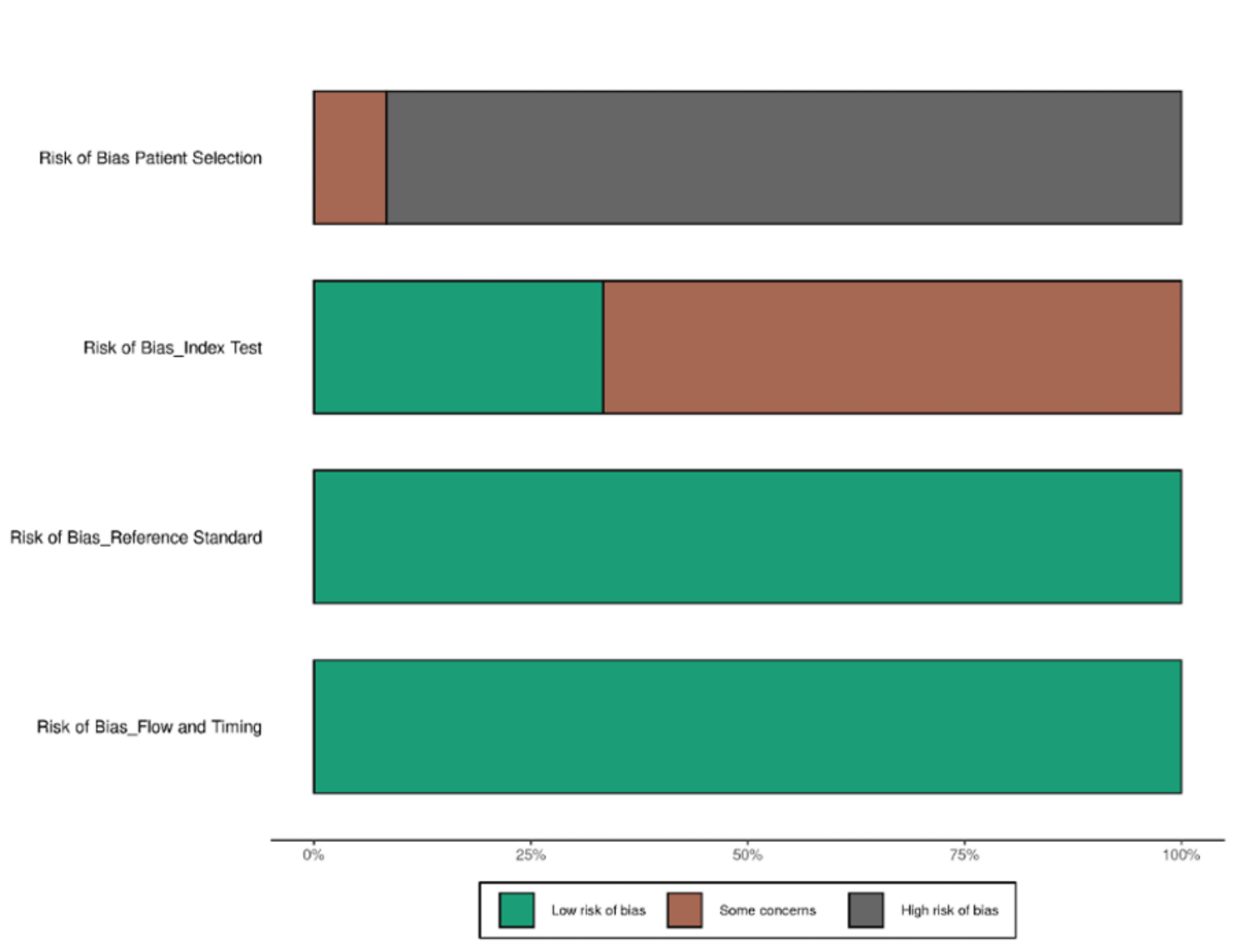
Risk of bias according to the Quality Assessment of Diagnostic Accuracy Studies 2 (QUADAS-2)

The reasons for bias were the lack of case-control, including patients with comorbidities, and inadequate description of thresholds in ML data sets. Figure 7 and Table 4 show the risk of bias and clinical applicability.

**Table 4:** Risk of bias and applicability of studies using QUADAS-2 QUADAS-2: Quality Assessment of Diagnostic Accuracy Studies 2

Author	Patient selection	Index test (machine learning)	Reference standard	Flow and timing	Risk of bias (patient selection)	Risk of bias (Index Test)	Risk of bias (reference standard)
Pecchia et al. [9]	Unclear	Yes	No	Yes	Unclear	High	High
Chen et al. [10]	Unclear	Yes	No	Yes	Unclear	High	High
Acharya et al. [11]	Unclear	Yes	No	Yes	Unclear	High	High
Acharya et al. [12]	Unclear	Yes	No	Yes	Unclear	High	High
Attia et al. [13]	Unclear	No	No	Yes	Low	High	High
Attia et al. [14]	Yes	No	Yes	Yes	Unclear	Low	Low
Hussain et al. [16]	Unclear	Yes	No	Yes	Unclear	High	High
Adedinsewo et al. [15]	Yes	No	No	Yes	Low	High	High
Attia et al. [17]	Unclear	No	No	Yes	Low	High	High
Harmon et al. [19]	Yes	Yes	Unclear	Yes	Unclear	Low	Low
Bachtiger et al. [20]	Unclear	Yes	No	Yes	Unclear	High	High
Attia et al. [18]	Yes	Unclear	No	Yes	Unclear	Low	Low
Surendra et al. [21]	Yes	Unclear	No	Yes	High	Low	High

Discussion

AI-based algorithms show high sensitivity and specificity compared to traditional ECG interpretation and methods used to detect HF. In particular, these models are precise and have a low FP rate that can help physicians promptly refer patients with abnormal ECGs, increasing the likelihood of identifying high-risk patients. In the last few years, there has been a surge in the development of new AI models employing deep learning techniques that show greater applicability than classical ML. A convolutional neural network trained using 12-lead ECG and echocardiogram data, including left ventricular ejection fraction called the AI-ECG model developed by Attia et al. has shown high clinical impact by its external and internal validation in multiple studies [13,14,17,20]. AI models have been designed to serve various purposes, such as initial assessment and screening [11,12,16,20,21] and remote health monitoring of patients, as described in a study [9].

These models can also be used in acute settings. Adedinsewo et al. created a model for use in emergency departments to identify patients with HF who present with dyspnea [15]. Acharya et al. proposed a technique that identifies patients with CHF with alarming (alerting) features, allowing clinicians to respond quickly [11]. These algorithms also show utility in predicting future adverse cardiovascular events in HF patients, facilitating risk stratification. De Michieli et al. showed that the patients evaluated for high-sensitivity cardiac troponin-T with positive AI-ECG for left ventricular systolic dysfunction had a higher risk of major cardiovascular events (MACE) [22]. Chen et al. have shown that ECG-EF can independently predict future CV adverse events [23].

The development of AI-ECG algorithms using single-lead ECG data in 2022 by Attia et al. has created an avenue for facilitating routine physical examinations [18]. This model, when applied to an ECG, enabled a digital stethoscope to reliably detect low ejection fraction, leading to easy screening, which is noninvasive and workflow-adapted [18,20]. The latest application of AI algorithm is seen in AI-ECG-enabled smartwatches that use digital ECGs and don’t require additional time-consuming input [21,24].

In a comparative study by Golany et al., the ML algorithm outperformed physicians in predicting LVSD from standard ECG, which shows that robust AI implementation in routine HF screening is the next step in future healthcare [25]. Despite formal quality assessment by QAUDAS-2, new tools are required to assess the quality of AI algorithms as these models don’t require patient recruitment and can be trained and tested on existing data sets. A retrospective design in early studies also risks selection bias since only high-risk patients who indicate ECHO and ECG are included. This can make the algorithm perform better than its accurate diagnostic and screening capabilities [6]. Inconsistency in the validation of algorithms also introduces bias with proper external validation of the AI model performed for the Attia algorithm only [14].

A well-known potential bias of AI-trained algorithms is that they are susceptible to errors when employed in a setting different from the training environment. This has previously limited the broad applicability of these algorithms. Harmon et al. showed that the AI-ECG network remained stable over time and across broad populations without retraining [19]. These models are generalizable across diverse patient populations, ethnicities, and age groups and can integrate among healthcare systems. However, continuous evaluation is still needed to see their performance over the years.

Future research is needed to predict the patterns of HF among the general population using these algorithms combined with other risk factors, biochemical markers such as natriuretic peptides, and demographic and socioeconomic factors. Prospective studies that assess reductions in HF hospitalization or all-cause mortality using AI algorithms compared to standard of care will lead to rational resource utilization [6]. Studies on patient perceptions of using ML algorithms for diagnosis must also be evaluated.

The performance of AI models in FP and FN rates compared to traditional ECG interpretation varies depending on the specific model and clinical setting. AI models, particularly deep learning-based CNNs, have demonstrated higher sensitivity in detecting subtle ECG abnormalities that might be missed by traditional ECG analysis, reducing FN rates in early-stage HF detection. However, FP rates remain a concern, especially in models trained on high-risk populations, as they may overdiagnose HF in general populations. In acute and emergency settings, AI-based ECG analysis enables rapid screening and triage of patients with suspected HF, but challenges such as real-time integration with clinical workflows, interpretability of AI decisions, and physician trust remain barriers to widespread adoption. The use of AI-ECG in smartwatches for real-world HF detection is promising, but its accuracy compared to clinical ECG and echocardiography remains under investigation, with concerns about signal quality, user adherence, and false alarms in nonclinical environments. The lack of external validation beyond Attia et al.’s algorithm raises concerns about generalizability, as models trained on homogeneous datasets may not perform well in diverse populations. Selection bias in training data, particularly underrepresentation of asymptomatic or ethnically diverse groups, may lead to algorithmic bias, limiting effectiveness in large-scale screening. Overcoming training-environment limitations requires continuous model refinement, inclusion of diverse datasets, and ongoing AI adaptation to real-world clinical conditions. The stability of AI-ECG models over time, as shown by Harmon et al., is a positive finding, but additional studies across different regions, ethnicities, and healthcare systems are needed to confirm reliability. Future research should prioritize randomized controlled trials (RCTs) assessing AI's impact on HF hospitalization and mortality, while cohort studies could evaluate long-term AI performance in clinical practice. Integrating biochemical markers (e.g., natriuretic peptides) and socioeconomic determinants into AI models could enhance predictive accuracy, but data standardization and ethical concerns regarding data privacy remain key challenges. The limitation of using only two databases and excluding studies with incomplete data may have restricted the comprehensiveness of this review, suggesting that future systematic reviews should incorporate more databases, gray literature, and unpublished data. The practical implementation of AI in daily clinical practice depends on clinician trust, interpretability, training requirements, and workflow integration. Concerns about AI replacing clinical judgment highlight the need for physician-AI collaboration rather than automation, while patient perceptions of AI-driven diagnosis warrant further exploration to address mistrust and ethical concerns regarding data transparency, liability, and algorithmic accountability in case of misdiagnosis.

Our review is conducted on a well-defined research question, and we have synthesized the current evidence; however, a potential limitation of our review is that we utilized two databases and studies with incomplete data were excluded. We conclude that AI-based models for HF detection are applicable diagnostic, screening, and monitoring tools in hospitals. They are cost-effective and ubiquitous and have strong sensitivity and specificity compared with the standard of care. These algorithms and other parameters like ECHO and biochemical profile help in early detection, prompt treatment, and prediction of adverse cardiovascular events in HF patients.

## Conclusions

Our review demonstrates that AI-based models for HF detection using ECG data exhibit high sensitivity and specificity, outperforming traditional ECG interpretation in many cases by detecting subtle abnormalities and reducing FNs. These models are cost-effective by enhancing diagnostic accuracy, reducing misdiagnosis, and potentially decreasing hospitalization rates through earlier intervention. AI’s ability to rapidly analyze large datasets, including ECG, echocardiographic parameters, and biochemical markers like natriuretic peptides, offers a more comprehensive assessment of cardiac function, aiding in risk stratification and personalized treatment. In real-world clinical settings, AI integration can streamline workflows by automating ECG analysis, supporting clinical decision-making, and improving efficiency in both hospital and outpatient settings. Beyond hospitals, AI has the potential to revolutionize primary care, emergency departments, and remote monitoring via smartwatches and wearable devices, allowing for continuous cardiac surveillance and early detection in high-risk populations. However, further research is needed to validate AI models across diverse patient populations, refine external validation methods, and ensure seamless integration into clinical workflows. As AI adoption expands, addressing data privacy, algorithmic transparency, and clinician acceptance will be crucial for maximizing its impact on cardiovascular care and improving patient outcomes globally.
